# World health by place: the politics of international health system metrics, 1924–*c.* 2010*

**DOI:** 10.1017/S1740022817000134

**Published:** 2017-11

**Authors:** Martin Gorsky, Christopher Sirrs

**Affiliations:** 1 Centre for History in Public Health, London School of Hygiene and Tropical Medicine, 15–17 Tavistock Place, London WC1H 9SH E-mail: martin.gorsky@lshtm.ac.uk; 2 Centre for History in Public Health, London School of Hygiene and Tropical Medicine, 15–17 Tavistock Place, London WC1H 9SH E-mail: christopher.sirrs@lshtm.ac.uk

**Keywords:** global health, international organizations, metrics, statistics, World Health Organization

## Abstract

This article examines the development of health system metrics by international organizations, exploring their relationship to the politics of world health. Current historiography treats measurement either as progressive illumination or adopts a critical stance, viewing indicators as instruments of global governance by powerful nations. We draw on diverse statistical publications to provide an empirical overview of change and continuity, beginning with the League of Nations Health Organization, which initiated health system statistics, and concluding with the *World health report 2000*, with its controversial comparative rankings. We then develop analysis and explanation of these trends. Population indicators appeared consistently owing to their protective function and compatibility with development thinking. Others, related to provision, financing, and coverage, appeared more sporadically, owing to changing trends and assumptions in international health. While partly affirming the critical literature, metrics were also used by peripheral or resistant actors to challenge or influence policy at the centre.

## Introduction

In the later twentieth century a language of ‘global health’ entered the lexicon of international policy-making. This usage signalled a qualitative difference between mid-century internationalism, founded on the shared interest of nation-states, and a new period in which the whole world was the canvas of action.^1^ Some key premises were that: infectious diseases observed no borders, while transmission risks had accelerated as travel patterns dissolved time and space; multinational corporations had diffused consumer appetites for tobacco, sugar, and fats, bringing pandemics of cancer and heart disease in their wake; telecommunications, then the internet, had immeasurably quickened the pace at which informatics were disseminated and debated; and established international bodies (principally the World Health Organization (WHO)) were largely superseded by new transnational actors, ranging from philanthro-capitalists to public/private initiatives, which were more dynamic if less accountable.^2^

Historians, including readers of this journal, will be cautious about the claims to novelty in headier articulations of this turn.^3^ If the essence of globalization is transnational connectedness, then it is reasonable to treat not only the integration of global markets but also the accompanying movements of populations, diseases, and medical cultures as very long-term processes.^4^ An ‘evolving international consciousness’ had first fostered a diplomacy addressing communicable disease in the early nineteenth century, and by the interwar period this had developed from brokering consensus over quarantines to installing regional surveillance networks.^5^ A global politics of the body is discernible at least from the 1920s, when racialized concerns with population health burst the bounds of colonial and national governance, to be taken up by the League of Nations Health Organization (LNHO) and the Office International d’Hygiène Publique.^6^ The long view has also sharpened critical perspectives, for example on how globalization has promoted the imperialism of biomedicine over other traditions, and has concealed post-colonial domination behind a language of universality.^7^ Historians’ engagement with ‘global health’ therefore aims both to delineate change from continuity and to illuminate the ways in which past trajectories have shaped the exercise of power.

The present article explores a central concept in this discourse: ‘health systems’ and the ways in which these have been conceptualized and measured. Though in lay terms simply synonymous with ‘health services’, the formal usage of ‘systems’ denotes something more: a holistic understanding of organized medicine, incorporating not only services but also labour force, financing, regulatory framework, patients, treatments, and health outcomes.^8^ The term and the associated concept gained currency from the 1960s, when authors began to develop typologies by which different arrangements could be classified, then to argue that comparison could drive cross-national policy learning.^9^ Health systems were thus conceived as dynamic models of inputs, processes, and outputs, which could be quantified to inform international studies, in the ongoing quest for equity, efficiency, and effectiveness.^10^ Perhaps the zenith of the approach came with the WHO’s *World health report 2000*, which ranked the health systems of 191 countries, digesting batteries of indicators into summary gradings representing criteria of fairness, responsiveness, and attainment.^11^ The results (which ranked France’s system highest and placed the United States at thirty-seven) generated controversy and angry national responses, though it also raised the political profile of health care questions.^12^

The heated debate which has attended such exercises arises also from broader concerns about the proliferation of indicators in global governance.^13^ This is held to be a recent phenomenon, synonymous with the era of globalization and the political dispensation accompanying it. Critical analysis extends far beyond health, to encompass quantification of areas such as economic behaviour, corruption, and ‘human development’.^14^ The key concern is that, although apparently consensual and transparent, statistical indicators are constructed by leading transnational actors to endow themselves with authority over weaker states in shaping policy and directing resources.^15^ Far from being neutral, they embody choices and values that may run counter to local democratic sentiment and other preferences, such as minimizing inequality. Illuminating the history of their construction and deployment is therefore a worthwhile task.^16^

Such contentions provide our starting point. Our first aim is corrective and empirical, in that we will show that the practice of collating health system statistics has a longer history than is widely assumed. We contend that, *avant la lettre*, the first envisioning and quantifying of international health systems came in the 1920s, in a series of LNHO yearbooks.^17^ It is at this point, and through organizations such as the LNHO, the Pan-American Health Organization (PAHO), and the Rockefeller Foundation, that ‘something approaching a global consciousness of health emerged’, with the LNHO becoming ‘the world’s leading purveyor of health information’.^18^ With dates loosely bounded by the first *Yearbook* in 1924 (published 1925) and by the *World health report 2000*, we document the development of these metrics. We will ask which organizations took responsibility, why they chose their subjects, and how they ensured common understandings. How, in short, were national health accounts ‘globalized’? Our second aim responds to the concern that indicators are always more than unmediated representations of reality, waiting to be uncovered. From their first appearance, health systems metrics not only revealed how other nations acted but also contained messages about how they could or should act. They were, in other words, discursive instruments, carrying either rhetorical force or, increasingly, power to direct policy-making. We therefore seek to uncover the political context in which international health statistics emerged, and the purposes that they have served. We begin, however, with a fuller examination of relevant literature.

## Historiography: metrics as empirical indicators for international comparisons

A limited literature discusses the technical history of health systems metrics, usually in introductory sections to contemporary cross-sectional analyses. Here the underlying assumption is broadly positivist, with indicators treated as self-evidently useful and desirable, and their history as one of progressive advance towards inclusion and accuracy. It suggests that their collection began meaningfully only in the later twentieth century, and, to the extent that explanation is offered, was driven by the rise in health costs from the mid 1970s and the ensuing concerns.

There is some allusion to forerunners in this writing, though these are passed over briefly. The first example of national health accounting (and by extension the basis of international models) is held to be the USA’s Committee on the Costs of Medical Care (CCMC) which in 1932 reported expenditure data for 1929: this contained an estimate of health spending as a proportion of national income, the composition and expenditure shares of the payees, socioeconomic distribution of sickness costs, and the nature and costs of services.^19^ The foundational documents of international comparison are considered to be two 1960s studies commissioned by the WHO from the British economist Brian Abel-Smith; the context of these is not described, only their role in harmonizing data.^20^ The other early influence is the United Nations’ general comparative national accounts, from 1953, which included a ‘satellite accounts’ approach applicable to health.^21^

Beyond this, explanation has concentrated on very recent factors. These include the cost-containment concerns of the 1970s, which are held to have inspired the Organization for Economic Co-operation and Development (OECD)’s Health Database, published from 1985, and the global comparisons undertaken in the early 1990s by the World Bank and PAHO.^22^ There was also the work of Eurostat, the statistical arm of the European Union, which, while carrying no brief for health policy, gave impetus through its freedom of movement principles to cross-national comparison and ideals of harmonization following the end of the Cold War.^23^ Outside the high-income countries, the general view is of early technical aspirations falling short due to poor data.^24^ Thus the annual reports of the International Monetary Fund (IMF) captured health as a proportion of government expenditure but excluded private sources. The World Bank’s 1993 *World Development Report* marked a major step towards a global picture, but it also revealed the weakness of data from countries lacking statistical capacity.^25^ In sum, such material provides helpful pointers to the institutions involved in the later twentieth century, but is otherwise mostly descriptive.

## Historiography: the use of indicators as instruments of global governance

In the last thirty years a substantial analytical literature has emerged which treats indicators not simply as representations of human action but also as ‘products’, which arise from political and institutional contexts and have active effects in the world. Sociologists and historians have long observed the need to study the construction of statistics, the unspoken assumptions underlying them, and their political impacts.^26^ Current interest follows the recent abundance of metrics in global politics, going beyond established fields such as productivity, health, and education to encompass many aspects of political and economic life. Alongside this have been a plethora of new agencies that generate such data, whose activities provoke reflection on metrics as tools of global governance, present and past.^27^

Much of this takes a newly critical perspective towards the power of numbers in today’s policy realm. Some highlight the competitive pressures of the ‘audit’ culture, others the forecasting errors perpetrated by credit-rating agencies.^28^ Many worry about the primacy in political discourse of gross domestic product (GDP), an indicator whose normative imperative of unceasing growth sits uneasily with environmental sustainability, and whose veracity for many low-income countries has been wildly overstated.^29^ Health metrics have figured somewhat in this critical literature, with respect both to the accuracy of those produced by poor countries and to their distorting effect on activity.^30^ For example, the muted success of the UN’s Millennium Development Goals for maternal health casts doubt on the suitability of a metrics-driven policy which neglects gender rights.^31^ Similarly, headline gauges of progress in areas such as sanitation or vaccination have obscured issues of local affordability and equity.^32^ This in turn raises the question of whether the indicators utilized by major donors to evaluate technocratic interventions may be distorting national policies and directing attention away from questions of poverty and inequality.^33^

Undergirding this critique of the contemporary rule of indices is a rich historiography which treats statistics as social construction.^34^ Poovey’s early history of numbering as a textual device demonstrates how the rhetoric of quantification lifted argumentation out of the realms of reasoning or ethical dispute. From the start, however, the apparent impartiality of ‘political arithmetic’ disguised the values and self-interest of protagonists.^35^ For Hacking the nineteenth century’s ‘avalanche of numbers’ lent a ‘veneer of objectivity’ to public policy-making.^36^ Yet the transposition of mathematical norms and probability onto the social world meant that projects to advance human betterment were henceforth intertwined with moralizing and class power.^37^ Also relevant is Foucault’s critique of vital statistics as instruments of control in a regime of ‘biopolitics’. His assertion was that a ‘medicalization’ of society had begun in the eighteenth century, entailing significant loss of individual liberties.^38^ A new politics emerged by which governing elites managed the ‘docile bodies’ of their populations to preserve human capital and protect themselves from infections.^39^ Statistics were the chosen technology of surveillance, delineating spaces of disease and segmenting by age to cultivate productive citizenry.^40^

Few scholars conclude that these critiques necessitate wholesale rejection of metrics as instruments of policy.^41^ Historically, ‘medical arithmetic’ produced a form of knowledge distinct from the ‘vague conjecture’ or ‘prejudices’ that went before, and Foucauldian pessimism pales against the human betterment achieved in areas such as environmental health and smallpox eradication.^42^ In medicine and other public realms such as engineering, quantification challenged the tacit knowledge legitimizing professional authority with rule-driven, transparent truth claims.^43^ Moreover, enumeration necessarily preceded political participation; hence, population statistics as electoral technologies were fundamentally equalizing.^44^ Indeed, the concern today is that indicators are democratically conceived rather than imposed, and that civil society should wrest back such processes of governance from the technocrats they empower.^45^

Whether critical or friendly, such analyses yield a range of conceptual propositions that help historicize the construction of numerical indicators. First, these serve a ‘branding’ or ‘flag-planting’ purpose, by which producer institutions assert jurisdiction over a particular field to underscore their own legitimacy.^46^ The very naming of an indicator stakes this claim to power, creating a lens through which the world may be viewed.^47^ Second, they simplify complexity, sharpening technical focus on an issue and rendering it amenable to cross-national comparison. Yet in this process they may disregard context and uncertainty, perforce excluding other potential areas of analysis.^48^ In achieving simplification, producers necessarily apply beliefs, judgements, and theories of change about the social world.^49^ Subsequently such choices can become reframed as entirely technical, yet they continue to incorporate ‘cognitive commitments of a powerful kind’.^50^ Third, they serve as tools of advocacy through their visual form. Numerical representation and the characteristic scalar rankings signify the imprimatur of scientific expertise and the articulation of fact rather than opinion.^51^ Such devices implicitly set standards and encourage governments or funders to evaluate performance and allocate resources accordingly.^52^ Contestation is rare and public scrutiny limited to first enunciation.^53^ For individuals they assert norms which may shape judgement about the world or personal behaviour.^54^ Finally, the stakeholders in the process may be delineated, the better to calibrate its power dynamics. There are ‘promulgators’ (those who commission and publicize indicators), ‘providers’ (the experts who create and maintain their supply), and ‘users’, including both the subjects of measurement and the states or funders that react to them.^55^

## Concepts, methods, summary results

We now turn to our empirical survey of health system indicators between 1924 and *c.* 2010. Although making some reference to national practices, we focus on statistics collected for international comparison and policy discussion. Again, we stress that, although the contemporary usage of ‘health system’ is discernible only from the 1960s, the assemblage of data juxtaposing financing, services, and population health somewhat predated this. These precursors both reflected and consolidated ideas about the interconnectedness of the different elements. For analytical purposes, we group the indicators of interest under the following main categories: inputs, processes, ‘outputs 1’ and ‘outputs 2’.

The category of ‘inputs’ covers different forms of financing, and in health accounting is sometimes referred to as ‘sources’. Total health expenditure is the fundamental comparative measure, usually expressed as a proportion of GDP or of gross national product (GNP). Other measures are public, private, or non-governmental health expenditure, typically formulated as a per capita sum, and more recently adjusted to US$ ‘purchasing power parity’. ‘Processes’, sometimes dubbed ‘uses’, covers areas of funded health activity, such as size of workforce, numbers of hospitals, beds, or primary care facilities, utilization rates of in- or out-patients, and vaccination rates. Metrics in the category ‘outputs 1’ calibrate the fairness and responsiveness of the system for users. Coverage measures include the proportion of the population with insurance or access to services. Responsiveness gauges individual users’ experiences through measures of participation, dignity, and satisfaction. Finally, ‘outputs 2’ incorporates general measures of population health, such as life expectation, mortality by age or cause, infant mortality, and morbidity, and more specific indicators of health system performance, such as amenable mortality and the disability adjusted life year (DALY).

Our data are drawn from a review of the annual statistical publications of several major international organizations. These begin in the interwar period with the LNHO, originating in the 1919 Treaty of Versailles and the provision in the Covenant of the League of Nations dealing with control and prevention of disease.^56^ Building on its Provisional Health Committee founded in 1921, the League’s Health Section was established under the Polish bacteriologist Ludwik Rajchman. It was supported by funding from US foundations and by an ‘internationally minded cadre of public health experts’, and its responsibilities included an International Service of Epidemiological and Health Statistics, whose remit was to ‘obtain, study and distribute information regarding diseases in different countries (including medical statistics)’.^57^ The other relevant agency from this period was the International Labour Organization (ILO), which had interests in social health insurance and undertook collaborative working with the LNHO, and later the WHO. Also descended from Versailles, the ILO was a European counterweight to Bolshevism, dedicated to industrial welfare and established under a French director, Albert Thomas.^58^ Its Social Insurance Section, led by Adrien Tixier, played a crucial role from the 1920s in promoting a European (Bismarckian) model of social security and collecting data to support its advocacy of standards on accident, medical, and sickness insurance.^59^

In the post-war era, the United Nations features in our survey owing to its inclusion of health measures within its extensive gathering of social and economic statistics. However, our main focus is the WHO, which became operational in 1948 as the UN’s specialized health agency, incorporating much of the LNHO’s work and some of its remaining staff.^60^ Its Constitution, adopted at the International Health Conference of July 1946, staked out a remit ostensibly beyond a narrow biomedical focus, with an articulation of health as ‘a state of complete physical, mental and social well-being’, and a fundamental human right. Among the WHO’s functions itemized in Article 2 of the Constitution were provision of ‘epidemiological and statistical services’, support for ‘strengthening health services’, and a ‘study and report’ role with respect to medical care ‘including hospital services and social security’.^61^

Finally, two agencies principally concerned with economic affairs are of importance. The World Bank was born out of the Bretton Woods Conference in 1944, initially to inject reconstruction loans into post-war Europe. It subsequently became the major international financial organization dedicated to development and poverty alleviation, comprising two relevant institutions: the International Bank for Reconstruction and Development and the International Development Association.^62^ Its interest in health dates to 1970, when its Population Projects Department began supporting health-related development projects. In 1980 it commenced direct lending for health purposes, believing that expertise in health programming would reduce poverty and increase productivity. The OECD similarly originated in post-war reconstruction, when, in 1948 the Organization for European Economic Co-operation was inaugurated to oversee the implementation of the Marshall Plan.^63^ Its successor, the OECD, was established in 1961 as a result of the 1960 Paris Convention with a mandate ‘to achieve the highest sustainable economic growth and employment and a rising standard of living in Member countries’. Nominally global in focus (currently with thirty-four members), the OECD primarily represented developed nations, exerting influence through its ‘global policy network’ of experts.^64^ As noted, its health initiatives date from the 1970s, and the concern with public expenditures articulated by its Secretariat and its Health Division, located in the Directorate of Employment, Labour and Social Affairs.^65^

We surveyed published statistical series and cross-sectional surveys generated by these organizations to construct a matrix showing the chronology of publication of particular categories and sub-categories of metrics. We have also undertaken archival research into the records of the LNHO, ILO, and WHO which allow us to contextualize and explore the production of indicators in fuller detail. This reveals that there have been numerous variations in the methodologies by which particular indicators were formulated over time. For example, the measurement by the WHO of morbidity from the 1950s was based on cases of notifiable diseases, until the mid 1980s, when statistics showing particular diseases, such as blindness, mental disorders, AIDS, or cancer were presented. Similarly, the standard gauge of service coverage in the WHO’s statistical annuals from the late 1950s was a ratio of health personnel to population, but from the 1980s this could include rates of maternity care or immunization. Our tabulation therefore presents broad categories, which each represent a constellation of closely related measures, rather than an absolutely uniform series.

**Figure 1:**
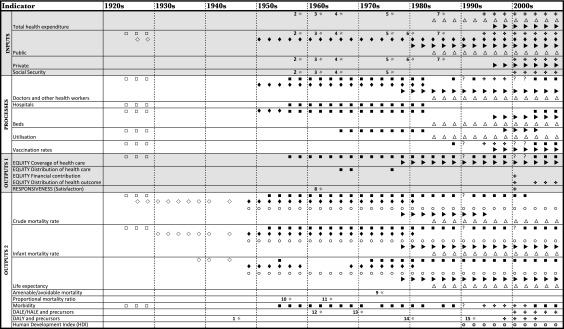
Health systems indicators published by international organizations, 1924–2000. **Notes:** Quarterly or monthly statistical publications (e.g. WHO World Health Statistics Quarterly) not included in survey. Indicator categories are for illustrative purposes only; they may relate to different phenomena. Statistics relate to year of publication, not dates covered by the data. Statistics published biannually may appear continuous. Inputs: health expenditures can be expressed in a variety of ways: in gross terms, per capita, or as a proportion of national income or expenditure (GNE, GNP, or GDP). Social security expenditure on health can be disambiguated from public/general government expenditure on health, or included within it depending on the analysis. Processes: With the exception of vaccination rates, processes refer to gross figures. Utilization refers to the movement of patients and the duration of disease: admissions and discharges, number of patient-days, bed occupancy rates, etc. Outputs 1: Coverage refers to proportionate figures of the above. These can be expressed as percentages (typically per 1,000 population) or ratios. Vaccination rates are thus included within coverage. Distribution of health care gives an idea about how health services and personnel are distributed spatially, e.g. between rural and urban areas, or between different regions or administrative districts of a country. Distribution of health outcome includes measures such as distribution of level of health across the population as a whole (measured in terms of the DALE/HALE); the burden of disease, disaggregated by age, sex, or mortality stratum (measured in terms of DALYs); or equality of child survival. Outputs 2: DALE/HALE and precursors denotes a constellation of related measures developed since the 1960s to weight life expectancies according to health/disease status. Sources: 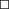 League of Nations International Health Yearbook (1925–1930) 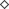 League of Nations Statistical Yearbook (1927–1945). Expenditure on public health identified in budget accounts for 1926 and 1927 (pub. 1927–8). 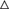 OECD Health Data File/Database (1985–) 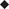 United Nations Statistical Yearbook (1948–) 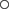 United Nations Demographic Yearbook (1949–) 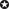 United Nations Development Programme, Human Development Report (1990–) 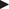 World Bank World Development Report (1978–): World Development Indicators; World Development Indicators (standalone volume) (1997–) 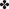 WHO World Health Report (1995–) 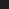 WHO Annual Epidemiological and Vital Statistics; World Health Statistics Annual; World Health Statistics (1952–) 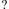 Data unavailable 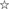 Miscellaneous reports and journal articles 1 Mary Dempsey, ‘Decline in tuberculosis: the death rate fails to tell the entire story’, *American Review of Tuberculosis*, 56, 1947, pp. 157–64. 2 ILO, *The cost of medical care*, Geneva: ILO, 1959. 3 Brian Abel-Smith, *Paying for health services: a study of the costs and sources of finance in six countries*, Geneva: WHO, 1963. 4 Brian Abel-Smith, *An international study of health expenditure and its relevance for health planning*, Geneva: WHO, 1967. 5 OECD, *Public expenditure on health*, Studies in resource allocation no. 4, Paris: OECD, 1977. 6 World Bank, ‘Health sector policy paper’, February 1980. 7 World Bank, *Financing health in developing countries: an agenda for reform*, Washington, DC: World Bank, 1987. 8 R. W. Revans, *Standards for morale: cause and effect in hospitals*, London: Oxford University Press, 1964. 9 David D. Rutstein et al., ‘Measuring the quality of medical care: a clinical method’, *New England Journal of Medicine*, 294, 11, 1976, pp. 582–8. 10 S. Swaroop and K. Uemura, ‘Proportional mortality of 50 years and above: a suggested indicator of the component “health, including demographic conditions” in the measurement of levels of living’, *Bulletin of the World Health Organization*, 17, 3, 1957, pp. 439–81. 11 Jan Drewnowski and Wolf Scott, *The level of living index*, Geneva: United Nations Research Institute for Social Development, 1966. 12 Barkev S. Sanders, ‘Measuring community health levels’, *American Journal of Public Health and the Nation’s Health*, 54, 7, 1964, pp. 1063–70. 13 D. F. Sullivan, ‘A single index of mortality and morbidity’, *HSMHA Health Reports*, 86, 4, 1971, pp. 347–54. 14 Ghana Health Assessment Project Team, ‘A quantitative method of assessing the health impact of different diseases in less developed countries’, *International Journal of Epidemiology*, 10, 1, 1981, pp. 73–80. 15 World Bank, *World development report 1993: investing in health*, Oxford: Oxford University Press, 1993.

Figure 1 shows the pattern of development of health system indicators across the organizations, between 1924 and the 2000s, going slightly beyond the data in the *World health report 2000*. In addition to the time series, the starred cells refer to one-off publications in which an early or embryonic appearance of a metric occurred. Some of these were in ILO or WHO documents, and some in academic journals. Some were eventually developed into time series, others were not.

Reading across from the left-hand to the right-hand columns, the overall pattern is as follows. The beginnings of comparative oversight in the 1920s were followed by a hiatus in the crisis decades of the 1930s and 1940s in all but basic mortality indicators. Revival between the 1950s and 1970s saw only limited interest in financing (Inputs), a clear concern with activity (Processes), and strong concentration on population health (Outputs 2). Advance towards the millennial profusion of metrics occurred from the 1980s, with the key differences being a broader view of financing, new outcome indicators which better captured morbidity effects, and some limited interest in equity and responsiveness (Outputs 1).

We can add detail to this account by considering the work of the different institutions, as denoted by the symbols. The first attempt at a holistic overview, incorporating metrics of financing, uses, and outcomes, was made by the LNHO. Its *Yearbook* series covered only the years 1924 to 1929, by which time thirty-seven countries were represented, though data formats were never standardized. Financial reporting took the form of governmental expenditure associated with preventive and curative health services, and numbers and membership of social health insurance schemes (which we treat as a crude indicator of coverage). Its functional successor, the WHO, produced no new financing series in its statistical annuals until the 1990s, although such information figured in its monthly statistics report (1968–1975), and the UN and later the World Bank did record public expenditure on health as reported in national accounts. Only in the 1990s was there an effort to create a comprehensive health costing series including private and voluntary sources, which had long been difficult to elicit. Occasional publications in 1959 (ILO), 1963, 1967 (WHO), and 1977 (OECD) calculated measures of national health expenditure as a proportion of national wealth; from 1985, the OECD produced a comparative health expenditure database for its member states, encompassing total and public expenditure. This was initially published in paper form (in 1985, 1990, 1993) before being disseminated electronically.^66^ However, the WHO did not adopt private expenditure on health as a continuous series until the 2000s, and has only recently established its Global Health Expenditure Database.

The main health system process measures have been of doctors, nurses, hospitals, beds, and patient utilization, expressed first as numbers, later as rates. These debuted in LNHO publications, disappeared with the cessation of the *Yearbook*, and then re-emerged in the WHO statistical annuals. There was some change at the end of the century, with discontinuation of recording of hospital and bed numbers, and the introduction of vaccination rates (rather than numbers), attended births, and various other indicators of utilization. Coverage measures were present from 1957, principally in the sense of population levels of physicians, but also in the WHO’s occasional attempts to capture distribution of health workforce by region and across urban/rural facilities (volumes published 1967, 1968, and 1976). Only in the 2000s did financial equity feature, as did responsiveness measures based on survey data of satisfaction and ‘dignity’ of treatment.

With respect to outcomes, two indicators of population health were present throughout most of the sequence: infant mortality rates and crude death rates, either at national level or by cause. These appeared not only in the LNHO *Yearbook* but also in the League’s statistical yearbooks, published 1927–45. Data on life expectancy were recorded from 1938, although WHO technical experts were concerned from the 1950s that these were not available for many low-income countries.^67^ Various other output indicators which sought a better linkage between health outcomes and services (as opposed to social or environmental determinants) appeared sporadically. The UN’s ‘proportional mortality ratio’ was trialled in the 1950s; this quantified deaths at age fifty or over as a proportion of all deaths, and was deemed appropriate for country comparisons of levels of development. Precursors to statistics which in different ways incorporated morbidity and mortality experience appeared from the 1940s, such as the Health Adjusted Life Expectation (HALE), the Disability Adjusted Life Expectation (DALE), the DALY, and ‘amenable mortality’ (deaths avoidable with timely access to health services). Of these, the DALY and DALE were later developed into time series, as ‘burden of disease’ measures.

In sum, while health system indicators in the era of globalization became more plentiful than in the past, their proliferation was another phase in a long history rather than a radical break. In contemplating Figure 1’s depiction of that history, questions present themselves. How do we explain disjunctures such as the break in the 1930s and 1940s, and the relative neglect of financing until the late twentieth century? What drove the continuities in uses and outcomes and the arrival of new variations in these fields? What accounts for the greater concentration of metrics evident from the 1980s? It is to these problems that we now turn.

## The politics of health systems metrics: national precursors

The arrival of international health statistics built upon national accounting work by bureaucracies of several advanced industrial economies to inform economic policy. The view that the American CCMC was the first attempt at a full set of national health accounts is broadly accurate. The measure of GNP was created in 1934 by the economist Simon Kuznets, and in wartime it was widely adopted by politicians, so only then was there a coherent benchmark against which national health activity could be set.^68^ The 1930s also saw an early British attempt to estimate total health expenditure (at ‘one-twenty-fifth of national income’) in a report by the think tank Political and Economic Planning.^69^ Probably other national cases would furnish more such examples. It is important particularly to note the role of Germany, whose developing statistical capacity after 1918 is well documented.^70^

Growing national experience with social administration also underpinned incipient international approaches. Germany’s sections of the LNHO *Yearbook* are notably more detailed on financing than most other countries, reflecting both its early start in administering social health insurance and its technical innovations in collecting macroeconomic data under the Weimar Republic. It was a German statistician, Emil Roesle, who was particularly instrumental at the LNHO in furthering the standardization of health and epidemiological data.^71^ German social security experts were also prominent at the ILO, where the Bismarck model of social welfare was regarded as pre-eminent, and their influence continued after 1933 despite Germany’s absence from the League of Nations.^72^

The use of scalar rankings to compare health inputs and processes by place also emanated from national practices. Taking the British case as an example, summary mortality tables by cause and place were published by the Registrar General of Births, Marriages and Deaths from the mid nineteenth century. In health policy rhetoric, the co-option of geographical variation data was discernible at least since Edwin Chadwick’s influential report of 1842.^73^ In addition, the annual publication of a battery of hospital statistics covering income, provision, utilization, and expenditure had been a staple of British public life since the 1890s.^74^ This arose initially from concerns about the performance of the voluntary hospital sector, with national tabulation begun to encourage institutional administrators towards emulating cost-effective practice elsewhere. Other national cases may yield similar forerunners.

## The politics of health systems metrics, 1924–46: the LNHO and ILO

The history of the LNHO’s statistics gathering and of its *Yearbook* is covered by Borowy, who notes *inter alia* the ‘branding’ function that this served.^75^ The newly created international body could justify its existence in part by its role as collector and disseminator of comparative health data, a process also discernible in its wresting of responsibility for the international classification of diseases (ICD) away from the rival International Statistical Institute.^76^ However, internal disagreement attended efforts to extend the *Yearbook*’s statistical remit beyond the realm of population health. This was opposed by more conservative national representatives such as Britain’s George Buchanan, who doubted their material benefits and refused to contribute until 1927. Britain’s reluctance may have reflected imperial unease, for nationalist movements were increasingly deploying arguments about colonial neglect of public health, and statistical amplification might have advanced their cause.^77^ Later, when the LNHO’s funding suffered drastic reduction following the 1929 recession, the yearbooks’ discontinuation was deemed an acceptable economy, though population health data continued to be published.^78^ This is unsurprising: epidemiological surveillance had been the original spur to intergovernmental ‘sanitary’ cooperation in the era of cholera and plague pandemics, and continued to form common ground of collaboration as the health effects of the depression were debated.^79^

In addition to the legitimation function, the ‘cognitive commitments’ of the main promulgators reflected a distinctly progressive ideal of European social medicine. Indeed, the sub-title of the *Yearbook* was ‘*reports on the public health progress*’ of participating countries.^80^ Exactly what constituted this forward march was signalled by the selection of material requested of respondent ministries, which, though varying across the period, began with population data (death rates and infant mortality), then dealt with recent legislation, followed by reports on prevention (sometimes differentiating infectious from social diseases), data on ‘curative medicine’ (hospitals, beds, workforce), and, from some countries, expenditures and social insurance fund details.^81^ As visual representations then, both providers and users were encouraged to conceive of health as a distinct arena of national life, whose prime goal was quantifiable improvement. This would be achieved by the growing role of the state, manifested in law-making, public spending, and public health administration. Just as protagonists of European social medicine envisaged the integration of preventive and curative facilities, bound together by welfare or insurance arrangements, so too did these statistical depictions of progress.^82^

Where did these beliefs of the LNHO promulgators come from? One source was collaboration between the LNHO and the ILO through the 1920s and 1930s on the relationship between social insurance and public health. The role of insurance schemes rose up the agenda as the interwar financial crisis intensified and state expenditures came under pressure.^83^ Activities of health insurance funds that conducted preventive activities raised issues of overlap and coordination. Thus, at the Sixth World Health Assembly in 1925, Czechoslovakia called for the LNHO to study the interface between public health administration and health insurance, which it had instituted in 1924.^84^ The result was the convening of a joint LNHO/ILO Expert Committee on Public Health Administration and Health Insurance to consider effective collaboration.^85^ Debate centred on ‘preventive results’ in fields relevant to both agencies, such as health education, maternal and child health, and tuberculosis control.^86^ Rajchman felt the activity ‘perhaps the most important yet undertaken’ by the LNHO, and, as support for extending social insurance rose during the Depression, calls grew for coordinating ‘committees of social medicine’ to oversee integration.^87^ Here was a preliminary articulation of international health system policy, emanating from a cadre of experts whose interests transcended national affiliations.

Finally though, despite this effort to conceive health holistically, the LNHO failed to embed a politics of ‘progress’ through international indicators. Instead they confronted the barriers to success, as *Yearbook* administrators discovered the incompatibility and unreliability of statistics, as well as clear differences in the enthusiasm of member states.^88^ Maximum participation was of thirty-seven states (in 1929), and, of all the reports made, 75% were from Europe; this was despite the League’s efforts, for example approaching sixteen Central and South American countries in 1927, but receiving no reply.^89^ The development of an international public health episteme was still rudimentary, there were significant variations in member governments’ statistical capacity, and language barriers made compilation difficult and expensive.^90^ Content was presented as a series of chapters on different states, with no publication of comparative rankings or uniform formats, such that country reports became patriotic accounts that fostered national prestige. Although the LNHO attempted from 1927 to harmonize tabulation through standardized forms, it failed to realize this before the *Yearbook*’s demise after 1929.^91^ Only in the late 1930s was a fresh attempt made to develop a comprehensive set of ‘health indices’, this time combining LNHO expertise with that of American New Deal reformers.^92^ However, this never left the planning stage and a long pause occurred before the original vision was revived.

## The politics of health systems metrics, 1946–72: the WHO and the ILO

Following the WHO’s formation, its *Annuals of epidemiological and vital statistics* began publication with the intent of acting as a ‘stimulant to improvement’ for member states.^93^ These continued the LNHO’s *Annual epidemiological reports* (1922–38), but were less detailed and experimental than the *Yearbook*, initially focusing on vital statistics and mortality by cause. Certain summary health data, such as numbers of beds and physicians, were presented in the UN’s general *Statistical yearbook* from 1950.^94^ However, only from 1957 did the WHO report health services data on hospitals, personnel, and vaccinations, expressed as crude numbers rather than comparable rates. Financial and coverage statistics were still absent.

What explains the abandonment of the broader vision espoused by the LNHO? Part of the answer lies with the reorientation of international health during the WHO’s early years. Advocates of social medicine were on the defensive after an initial effort to integrate social security into the WHO’s remit was rebuffed.^95^ Instead, resources and effort were directed to biomedical initiatives such as the Global Malaria Eradication Programme and mass immunization against smallpox.^96^ Underlying this was a faith in technological solutions, coupled with a philosophy of development that espoused vertical interventions by high-income countries.^97^ Cold War politics also underlay the United States’ aversion to engagement with ‘socialized medicine’ – an inflammatory issue in its own domestic politics. Thus concern with insurance coverage largely dropped off the international health agenda, and no influential lobby existed to advocate reconnecting analysis of health financing to the now marginal areas of health planning and strengthening.

Compounding this lack of political will were the methodological and conceptual difficulties surrounding the standardization of measures of utilization and provision. The WHO’s Expert Committee on Health Statistics was aware from 1950 that such data were needed to ‘provide a basis for future planning’ that was ‘effective and economical’.^98^ However, common definitions of basic units of analysis were the prerequisite of comparability and in the 1950s the main focus of effort was instead the seventh revision of the ICD.^99^ Also in line with the broader UN mission of the 1950s came the UN’s proportional mortality ratio, a metric aimed at ranking poor countries on a scale of development. It was not until 1963 that the Expert Committee officially recommended the routine international collection of hospital data (including bed, patient, and personnel statistics), to complement mortality and morbidity data and inform the administration of a nation’s ‘general health programme’. Broken up and renamed the WHO *World health statistics annual* from 1965, the third volume of the WHO’s yearbook was dedicated to statistics of health establishments and personnel.^100^

Behind this upgrading of health indicators in the 1960s was a new interest in system planning, driven not from the centre but from the regions of the WHO, and particularly Latin America. As Carlos Luis González, a Venezuelan professor of preventive and social medicine argued in 1966, in many countries the health statisticians’ operational role was poorly understood and divorced from health policy; thus the statistician was ‘in the position of a pilot required to steer a ship without knowing where it is going’.^101^ This *cri de coeur* reflected efforts to professionalize planning led by officials from Chile, Columbia, and Venezuela, alongside various American universities and the Milbank Memorial Fund. The resulting ‘PAHO/CENDES’ planning frame, which sought to model population health needs and then shape provision accordingly, was one among several methods adopted by the WHO, which also engaged with some post-independence African states to devise ‘national health plans’.^102^ In 1967 its Expert Committee on National Health Planning in Developing Countries, chaired by the Indian health official Nowshir Jungalwalla, lamented ‘the lack of accurate and complete data’ for health planning.^103^ By 1969 the intellectual case for data on personnel, facilities, and health services organization to guide this process was accepted at the WHO.^104^ Between 1967 and 1972, therefore, statistics on epidemiology (outputs) and health services (uses) began to come together in a more coherent framework, alongside institutional changes that culminated in the creation of a Division of Strengthening of Health Services.^105^

Health financing still remained sidelined, however, owing to the WHO’s long-standing reluctance to engage with the subject of social security. Despite this the torch was kept alight by groups within the ILO and the WHO, who began resolving technical questions of standardization but failed to initiate a continuous series. In the 1950s, following the failure of the social medicine lobby at Geneva to secure a progressive convention on health and medical insurance, the ILO had begun research on national health financing.^106^ A report appeared in 1959, authored by Laura Bodmer, an ILO official, which compared health costs under social security across fourteen countries for 1945–1955, finding them broadly stable.^107^ The political implication was that expansive public programmes under NHS or ‘Bismarck’ models were entirely affordable, while comparison with the United States revealed that they were no more costly than ‘care privately obtained’.^108^ Next came two WHO studies by Brian Abel-Smith, one of the first international experts to calculate health expenditure as a proportion of GNP.^109^ First, in a six-country survey of 1963, Abel-Smith developed a comparative methodology based on standard definitions of ‘health services’, their cost headings, and the national accounting measures against which to set them.^110^ A more ambitious survey in 1967 applied this method to thirty-three nations, demonstrating empirically that the wealthier countries with better health indicators spent most, and vice versa.^111^ As before, the political subtext was to defend public systems, for, now that a secular upward trend in health spending was discernible among industrialized nations, it appeared that those with larger private sectors were less cost-effective.^112^

The intellectual trail leading to these initiatives again goes back to Latin America, and to an influential WHO discussion paper by Hernan Romero, Professor of Hygiene and Preventive Medicine at the University of Chile.^113^ Noting the issue of rising costs worldwide and uncertainty about how added health expenditure translated into improved health, Romero urged that the ‘bases of wholesome medical economy are laid down’.^114^ Chile’s case is suggestive of why voices from the periphery were less inhibited about placing health financing on the international agenda. It was an early adopter of Bismarkian welfare, establishing a sickness insurance system for blue-collar workers in 1924–25, and for white-collar workers in 1938, followed by a comprehensive national health service in 1952, covering about 60% of Chileans.^115^ Thus questions of how best to manage a pluralistic health economy, with ambitions to enhance coverage under a NHS model, loomed large for Chilean social medicine.^116^ Yet, despite regional calls and Abel-Smith’s methodological innovations, financial indicators still did not become a routine element of statistical reporting. Political sensitivities are the probable explanation.

## The politics of health systems metrics, 1972–2000: the West

The eventual arrival of consistent and comparable data on health financing occurred in the 1970s, but was limited to the advanced industrial nations. Why did this happen? Beyond the Abel-Smith and Bodmer exercises, the other precursor was the System of National Accounts (SNA) developed in the UN after 1947, whose national data also recorded subsidiary items such as public health, defence, and education.^117^ Publication began in 1953, continuing in the UN *Yearbook of national accounts statistics* (1957–82).^118^ Such broad-brush categories were of limited use to medical administrators as the intention was to describe general economic activity, rather than to detail financial flows for health policy-making.^119^ Not until the 1993 SNA revision was more detailed information on health systems financing introduced, with the promotion of ‘satellite health accounts’ that expanded on the core national accounting framework and were adopted by a few countries, such as France.^120^

Comparative data from the UN were therefore limited when the OECD took up the mantle in 1977.^121^ This was not through an interest in health *per se*. Rather, the promulgators were responding to rapid public sector growth across OECD countries, and its economic policy implications; companion studies tackled air pollution, education, and income maintenance programmes. As of 1977, health expenditure accounted for approximately 4.5% of GDP on average, an increase of almost one-half since 1965, and this forced discussion of efficiency and cost-effectiveness onto the agenda.^122^

Thus it was the sense of escalating concern among the Western economies, in the aftermath of energy price rises and the ending of the *trentes glorieuses*, that finally galvanized the integration of financial data.^123^ Having produced telling findings that, for example, variations in the hospital cost ratio accounted for over half of all differences between countries, officials in the OECD Secretariat developed a large and more consistent international dataset.^124^ A 1985 report, *Measuring health care 1960–1983*, contained over seventy comparative tables covering spending, utilization, and delivery, extending back to 1960 for some countries, and laying the groundwork for an OECD health database, eventually distributed electronically via diskette. Emphasis was placed on how money was used, rather than its sources, since most OECD members operated social insurance or general taxation models, and this consolidated the idea of a unitary health system with discrete inputs, outputs, and uses; another upshot was the realization that accurate evaluation required an outcome indicator that captured the health system contribution better than general mortality.^125^ Academic efforts to provide this through indices of ‘amenable mortality’ were under development, as were gauges of morbidity expressed through the quality-adjusted life year (QALY), but neither provided a continuous series. In 2000, a fuller uniform system of health accounts was developed by OECD officials, and its main architect, Jean-Pierre Poullier, subsequently moved to the WHO in Geneva. The 2011 revision of the uniform system by the OECD, Eurostat, and the WHO now underpins the WHO’s Global Health Expenditure Database, which details health expenditure in all WHO member countries.^126^

Ironically, then, it was not the calls of development planners or the emergent welfare states which consolidated the final arrival of comprehensive health metrics by 2000. Instead, it was the contradiction between rising consumer demand for health care and the limits to public financing in the West which did so. As a 1987 OECD report observed: ‘With the achievement of almost universal access in most countries, efficiency and effectiveness issues have moved to the forefront of the policy debate.’^127^ The comment about universalism did not, of course, apply to the Global South.

## The politics of health systems metrics, 1968–2000: the world

The end of the twentieth century saw the extension to the global canvas of this broader range of health system indicators, as well as the emergence of new outcome measures that integrated mortality with non-fatal diseases. As Figure 1 suggests, only at the millennium was the LNHO’s original aspiration of comprehensiveness fully realized, though the inspiration was no longer ‘progress’ through extending public provision. Instead, it followed a series of changes in the composition of global health institutions, which brought concomitant shifts in the politics of development. Among the promulgators, the World Bank assumed a much greater role, and health economics (or at least its language and conceptual frame) became the dominant discipline among the data providers.

How did this come about? International health policy had moved away from vertical interventions in the 1970s as the limits of malaria control through pesticides and chloroquine became apparent. Instead, under the WHO Director-General Halfdan Mahler, a policy of ‘Health for All’ through service strengthening was espoused. The 1978 Alma Ata Declaration set out the goal of universalizing access to primary health care by working with indigenous providers to deliver services, on the model of China’s barefoot doctor approach, among others.^128^ This reorientation reflected a relative decline of Western power at the WHO, conceded in part to the Soviet Union and in part to more assertive nations of the Global South, now emerging from colonial subjugation. It also paralleled the shift in economic policy in which poorer nations rejected trade and loan arrangements that arguably perpetuated ‘under-development’ for the South while enriching the West. Now they wanted lending and aid geared to building domestic productive capacity, particularly through import substitution.

If this turn was not obviously influential on policy towards metrics, the reaction to it certainly was. By the 1980s the march towards a new international economic order was faltering as debt crises consumed various nations of Africa and Latin America. The IMF applied more stringent conditionality on further borrowing, including ‘structural adjustment’, which entailed reduced public expenditure on social purposes. The United States also grew impatient with the WHO, resenting positions adopted on issues such as infant formula milk and drug marketing, which it believed inimical to its interests.^129^ Henceforth it reined back its financial subscriptions and lent support to the growing leadership role of the World Bank in health financing. In these circumstances the goal of universal primary care came under question, and players such as the World Bank and UNICEF instead embraced ‘selective primary health care’.^130^ Aspirations would be pared back and resources concentrated on programmes whose efficiency and effectiveness could be convincingly demonstrated.

These events provided the intellectual context for the new global health indicators. A key influence was the *World development report 1993*, in which the World Bank articulated its vision of health system strengthening and proffered a new indicator of ‘global burden of disease’ (GBD), through which its efficacy could be measured.^131^ The aim of the metric was to inform policy-makers of the ‘relative magnitude’ of the different diseases affecting their populations.^132^ This would improve allocative efficiency, allowing prioritization of ‘cost-effective programmes that would help the poor’, and also provide a sound gauge for evaluating interventions.^133^ Accompanying this was the Bank’s advocacy of greater pluralism in financing, based on its long-standing preference for non-governmental insurance and commercial user fees.^134^ Standard indicators therefore began to disaggregate expenditure components into governmental, private, and out-of-pocket sources. Such categories reflected the reality that all health systems draw on mixed sources of income. Yet they also endorsed a new dominant ideology, in which expenditure on health services was seen not as a public good but as essentially discretionary, and thus without a firm justification for the state’s dominant role.^135^

Considerable debate has attended the new metrics which underpinned GBD studies, DALYs and DALEs. Crudely, DALYs express years of healthy life lost due to morbidity and premature mortality – a ‘health gap’ – while DALEs quantify expected life years in full health – a ‘health expectancy’.^136^ Although sometimes regarded as a recent ‘economicizing’ of health bound to global capital’s ‘regimes of sovereignty’, these have a distant progeny.^137^ LNHO experts had mooted a ‘single health index’ in 1937, before dismissing it as likely to obscure the ‘individuality of local problems’ (this would become a recurrent critical trope).^138^ Other precedents devised variously by epidemiologists, psychiatrists, operational researchers, and economists include: the UN proportional mortality ratio (1957), described above; the PAHO/CENDES planning method (1965), which had assumed that weighting for morbidity was impossible; Sanders’ ‘productive man-years’ gauge (1964), which measured health system efficacy according to human capital criteria; Torrance’s utility rankings of different health states (1970); Sullivan’s concept of disability-free life expectancy (1971); Rosser’s scalar indices of the ‘disutilities’ associated with different morbidities (1972); and finally Zeckhauser and Shepard’s ‘quality adjusted life year’ (1976), a composite mortality/morbidity index which gained wide acceptance.^139^ Building on these, the DALY/DALE calculations used panel data to weight the gravity of disability across different disease groups, a sex adjustment to correct for greater female longevity, and an age weighting that valued the lives of productive adults over younger or older groups.^140^

The DALY was an indicator which attracted much comment, but whose authority was quickly established and normalized.^141^ To what extent does the surrounding debate validate the criticisms voiced by opponents of ‘governance by indicators’? Some of the objections were pragmatic. Was it legitimate to focus on diseases, when cost-effective policies hinged on the evaluation of interventions and technologies? Given multi-pathologies and co-morbidities, how could a DALY even be meaningful?^142^ Others were methodological. How valid were the processes followed to estimate disability weights, which relied on technocratic surveys? Others examined the underlying ethical and political judgements: an equity adjustment had been introduced to avert results which valued the lives of older Westerners higher than those of citizens of countries with low life expectancy, but this produced artificial results and failed to address income and socioeconomic inequalities.^143^ Others condemned the enterprise for monetizing human life, rendering health development aid as investment in productivity.^144^

The DALY’s providers trenchantly defended their measure, however.^145^ For them, it was a transparent metric which lifted policy debate and resourcing decisions out of the realm of special interests, provided a gauge for analysing cost-effectiveness over time, and facilitated comparative judgement on health systems. It incorporated morbidity data, an advance on earlier approaches limited to mortality, and thus illuminated hitherto invisible patterns – the scale of mental illness, for example. The lack of an adjustment for social inequality was ethically defensible on the grounds that all lives were valued equally, while the weighting in favour of productive lives simply acknowledged an ‘apparently widely held preference’.^146^ Nor did DALYs privilege private over statist health systems, instead providing a tool for public officials to hold insurers and market providers to account. The role of GBD evidence in Mexico’s introduction of universal coverage illustrates their usefulness to progressive politicians.^147^ On balance, the critique of the DALY is not yet compelling, and awaits more sustained evaluation of its impacts on global health.

A similarly ambiguous assessment attends the health systems rankings in the *World health report 2000*, which included the DALE. The chorus of criticism that this received on publication would have gladdened any metrics sceptic. To some the exercise cloaked an ideological commitment to marketized health care, defining ‘systems’ to exclude environmental determinants of health inequalities, and marginalizing community-based primary care or public health interventions.^148^ Conversely, marketeers discerned a Marxist ideological bias, presumably because the US system ranked so poorly.^149^ Others concentrated on technical weaknesses such as the limited conceptualization of system inputs, or the fragmentary underlying data, where heroic estimation procedures were used to fill gaps (some 60% of data points).^150^ Arguably, the whole enterprise was illegitimate, for why should national health systems be expected to conform to some common performance criteria?^151^

With time, however, a more moderate judgement has emerged, concentrating on the *Report*’s rhetorical and performative impacts. The 2003 Mexican reforms noted above, which improved coverage through higher and more redistributive expenditure, were justified in terms of that country’s poor ‘fairness’ rankings.^152^ Similarly, the Chinese leadership’s embarrassment over its lamentable position (188th) in the equity table inspired the revival of rural cooperative medical insurance from 2002.^153^ More broadly, the United States’ failures in comparative rankings were a staple argument in the debates preceding the introduction of ‘Obamacare’ in 2010.^154^ In addition to placing health system reform onto political agendas, the *Report* stimulated a busy research programme seeking to improve data on outcomes and responsiveness.^155^ Notwithstanding the consensual and market-friendly language in which it was couched, it also helped rehabilitate the activist state as ‘steward’ of its citizens’ health.^156^

## Conclusion

We have argued that today’s global health systems indicators have a long history which is worth recovering. Partly this is to bring empirical accuracy to an area often poorly documented, and to clarify the organizational and technical context in which these statistics were gathered. Partly it is to consider health metrics as time-bound constructs, relating their emergence and usage to the changing politics of international health. After categorizing some key areas for analysis, we charted their presence over time, before considering reasons for adoption and persistence.

We found an early aspiration to comprehensive, comparative metrics on the part of the LNHO, which in the 1920s sought to bring together details on financing, services, and mortality, and to document and proselytize for ‘progress’ in social medicine. However, failure to standardize many of the requisite measures, and the shortfalls of resources and members’ support, left ambitions largely unfulfilled. Subsequently a consensus was reached over the importance of population health statistics, as the foundation stone of intergovernmental collaboration. Historically, a defensive national interest had been their original source, but under the early WHO such numbers became tools for gauging pace of ‘development’ in an era of vertical health programmes. Health services metrics were also published continuously by the WHO, though initially these were relatively crude.

Gradual movement towards greater sophistication gathered pace in the late 1960s, as ideas about health planning gained traction. The drivers for this were both at the centre, where the end of empire opened new prospects for international intervention as a development project, and in the periphery, where incipient welfare states sought better data for policy learning. The missing element remained comparative financial indicators. Their absence was a legacy of the founding conviction that health funding policy was a matter for national judgement, and thus, implicitly, that quantification of public spending signalled its advocacy and should be disavowed. Sporadic efforts at standardization in the 1950s and 1960s provided a bridge with the social medicine traditions, but only with the fiscal crisis of Western welfare states did sustained support for continuous time series of health financing emerge. At the end of the century the holistic quantification of health systems, capturing inputs, processes, and outcomes, which was pioneered in the West, was extended worldwide. By 2000 this had placed the relationship between health improvement and equitable systems financing at the centre of global policy discourse. Continuing fragility in poor countries was, however, repeatedly exposed by crises such as those of HIV/AIDS and Ebola. Whether the inclusion of universal health coverage among 2015’s Sustainable Development Goals will herald the reuniting of health system metrics with the social medicine tradition from which they sprang remains to be seen.

## Footnotes

1

BrownTheodore, CuetoMarcos, and FeeElizabeth, ‘The World Health Organization and the transition from international to global public health’, American Journal of Public Health, 96, 1, 2006, pp. 62–72 1632246410.2105/AJPH.2004.050831PMC1470434.

2

HanefeldJohanna, ed., Globalisation and health, Maidenhead: Open University Press, 2015 ; BirnAnne-Emanuelle, PillayYogan, and HoltzTimothy, Textbook of international health: global health in a dynamic world, Oxford: Oxford University Press, 2009, pp. 6–8 , 417–69.

3 Birn, Pillay, and Holtz, *Textbook of international health*, p. 8.

4

HarrisonMark, ‘A global perspective: reframing the history of health, medicine, and disease’, Bulletin of the History of Medicine, 89, 4, 2015, pp. 641–643 10.1353/bhm.2015.0116PMC489865726725408.

5

HarrisonMark, ‘Disease, diplomacy, and international commerce: the origins of international sanitary regulation in the nineteenth century’, Journal of Global History, 1, 2, 2006, p. 216 ; SealeyAnne, ‘Globalizing the 1926 International Sanitary Convention’, Journal of Global History, 6, 3, 2011, pp. 431–455 .

6

NegriAntonio and HardtMichael, Empire, Cambridge, MA: Harvard University Press, 2000, pp. 22–30 ; BashfordAlison, ‘Global biopolitics and the history of world health’, History of the Human Sciences, 19, 1, 2006, pp. 67–88 .

7

AndersonWarwick, ‘From subjugated knowledge to conjugated subjects: science and globalisation, or postcolonial studies of science?’ Postcolonial Studies, 12, 4, 2009, pp. 389–400 ; HodgesSarah, ‘Second opinion: the global menace’, Social History of Medicine, 25, 3, 2012, pp. 719–728 2634546910.1093/shm/hkr166PMC4558944.

8

MillsA. J. and RansonM., ‘The design of health systems’, in M. H. Merson, R. E. Black, and A. J. Mills, eds., International public health: diseases, programs, systems, and policies, Gaithersburg, MD: Aspen Publishers, 2001 .

9

AndersonO. W., ‘Medical care: its social and organizational aspect. Health service systems in the United States and other countries’, Parts I and II, New England Journal of Medicine, 269, 16, 1963, pp. 839–843 1405226810.1056/NEJM196310172691605, and 269, 17, 1963, pp. 896–900; RoemerM. I., Health care systems in world perspective, Ann Arbor, MI: Health Administration Press, 1976 .

10

NavarroVicente, ‘Planning personal health services: a Markovian model’, Medical Care, 7, 3, 1969, pp. 242–249 579899910.1097/00005650-196905000-00008; AndersonO. W., Health care: can there be equity? The United States, Sweden, and England, New York: Wiley and Sons, 1972 ; KohnRobert and WhiteKerr, eds., Health care: an international study, Oxford: Oxford University Press, 1976 .

11

World Health Organization, The world health report 2000. Health systems: improving performance, Geneva: WHO, 2000 .

12

McKeeMartin, ‘The *World health report 2000*: 10 years on’, Health Policy and Planning, 25, 2010, pp. 346–348 2079812610.1093/heapol/czq032.

13

AdamsVincanne, ed., Metrics: what counts in global health, Durham, NC: Duke University Press, 2016 .

14

DavisKevin, FisherAngelina, KingsburyBenedict, and MerrySally Engle, eds., Governance by indicators: global power through quantification and rankings, Oxford: Oxford University Press, 2012 .

15

DavisKevin, KingsburyBenedict, and MerrySally Engle, ‘Indicators as a technology of global governance’, Law and Society Review, 46, 1, 2012, pp. 71–104 .

16

AdamsVincanne, ‘Metrics of the global sovereign’, in Adams, Metrics, pp. 19–54 .

17

BorowyIris, Coming to terms with world health: The League of Nations Health Organisation 1921–1946, Frankfurt: Peter Lang, 2009, pp. 177–183 ; BorowyIris, ‘World health in a book: the *International health yearbooks*’, in Iris Borowy and Wolf Gruner, eds., Facing illness in troubled times: health in Europe in the interwar years 1918–1939, Frankfurt am Main: Peter Lang, 2005, pp. 85–128 .

18 Harrison, ‘Global perspective’, p. 671; Patricia Anne Sealey, ‘The League of Nations Health Organisation and the evolution of transnational public health’, PhD thesis, Ohio State University, 2011, p. 202.

19

FetterBruce, ‘Origins and elaboration of the National Health Accounts, 1926–2006’, Health Care Financing Review, 28, 1, 2006, pp. 53–54 PMC419497217290668; Committee on the Costs of Medical Care, Medical care for the American people: the final report of the Committee on the Costs of Medical Care, Chicago, IL: University of Chicago Press, 1932 , ch. 1.

20

MurrayC. J. L., GovindrajR., and MusgroveP., ‘National health expenditures: a global analysis’, Bulletin of the World Health Organization, 72, 4, 1994, pp. 623–637 7923542PMC2486604.

21

BermanPeter, ‘What can the U.S. learn from national health accounting elsewhere?’, Health Care Financing Review, 21, 2, 1999, pp. 48–49 PMC419465211481785.

22

SchneiderMarkus, ‘National health accounts: a tool for international comparison of health spending’, in T. R. Marmor, R. Freeman, and K. H. G. Okma, eds., Comparative studies and the politics of modern medical care, New Haven, CT: Yale University Press, 2009, pp. 319–345 .

23

van MosseveldC. J. P. M., International comparison of health care expenditure, existing frameworks, innovations and data use, Voorburg: Statistics Netherlands, 2003, pp. 3–5 .

24

PoullierJean-Pierre, ‘Administrative costs in selected industrialized countries’, Health Care Financing Review, 13, 4, 1992, pp. 167–173 10122004PMC4193256.

25 Berman, ‘What can the U.S. learn?’, p. 50.

26

StarrPaul, ‘The sociology of official statistics’, in W. Alonso and P. Starr, eds., The politics of numbers, New York: Russell Sage Foundation, 1987, p. 8 .

27

CooleyAlexander, ‘The emerging politics of international rankings and ratings’, in Alexander Cooley and Jack Snyder, eds., Ranking the world: grading states as a tool of global governance, Cambridge: Cambridge University Press, 2015, pp. 1–38 .

28

PowerMichael, The audit society: rituals of verification, Oxford: Oxford University Press, 1997 ; FioramontiLorenzo, How numbers rule the world: the use and abuse of statistics in global politics, London: Zed Books, 2014, pp. 39–67 .

29

FioramontiLorenzo, Gross domestic problem: the politics behind the world’s most powerful number, London: Zed Books, 2013 ; MorseStephen, Indicators and indices in development: an unhealthy obsession with numbers, London: Earthscan, 2004, pp. 33–59 ; JervenMorten, Poor numbers: how we are misled by African development statistics and what to do about it, Ithaca, NY: Cornell University Press, 2013 .

30 See the contributions to Adams, *Metrics*.

31

YaminAlicia Ely, ‘Sexual and reproductive health, rights, and MDG 5: taking stock, looking forward’, in Malcolm Langford, Andy Sumner, and Alicia Ely Yamin, eds., The Millennium Development Goals and human rights: past, present and future, Cambridge: Cambridge University Press, 2013, pp. 232–254 .

32

LangfordMalcolm, ‘Rethinking the metrics of progress: the case of water and sanitation’, in Langford, Sumner, and Yamin, Millennium Development Goals, pp. 461–483 ; FisherAngelina, ‘From diagnosing under-immunization to evaluating health care systems: immunization coverage indicators as a technology of global governance’, in Davis et al., Governance by indicators, pp. 217–246 .

33 Fioramonti, *How numbers rule*, pp. 144–91; BirnAnne-Emanuelle, ‘Gates’s grandest challenge: transcending technology as public health ideology’, Lancet, 366, 2005, pp. 514–519 1608426110.1016/S0140-6736(05)66479-3.

34 Cooley, ‘Emerging politics’, pp. 17–18.

35

PooveyMary, The history of the modern fact: problems of knowledge in the sciences of wealth and society, Chicago: University of Chicago Press, 1998, pp. xiii–xviii , 40–59, 120–38.

36

HackingIan, The taming of chance, Cambridge: Cambridge University Press, 1990, pp. 3–4 .

37
*Ibid.*, pp. 34–7, 47–54, 76, 118–20, 163–9.

38 Michel Foucault, ‘The crisis of medicine or the crisis of antimedicine?’, lecture given at the State University of Rio de Janeiro, Brazil, 1974, trans. E. Knowlton and C. O’Farrell, *Foucault studies*, 1, 2004, p. 6; FoucaultMichel, ‘The politics of health in the eighteenth century’, trans. Colin Gordon, in Michel Foucault, Power/knowledge: selected interviews and other writings 1972–1977, New York: Pantheon Books, 1980, pp. 166–182 .

39

FoucaultMichel, *The history of sexuality*. *Volume I: an introduction*, trans. Robert Hurley, New York: Pantheon, 1978, pp. 140–141 ; Foucault ‘Politics of health’, pp. 172–7.

40

FoucaultMichel, ‘The birth of social medicine’, trans. Colin Gordon, in Foucault, Power/knowledge, pp. 134–156 .

41 Fioramonti, *How numbers rule*, pp. 195–7.

42

RusnockAndrea, Vital accounts: quantifying health and population in eighteenth-century England and France, Cambridge: Cambridge University Press, 2002, pp. 10, 67, 92–106 , 152 (quoting James Burges, *An account of the preparation and management necessary to inoculation*, London, 1754, and William Heberden, *Observations on the increase and decrease of different diseases*, London, 1801).

43

MatthewsJ. Rosser, Quantification and the quest for medical certainty, Princeton, NJ: Princeton University Press, 1995, pp. 143–145 ; PorterTheodore, Trust in numbers: the pursuit of objectivity in science and public life, Princeton, NJ: Princeton University Press, 1995 10.1177/03063129902900400711623934.

44 Rusnock, *Vital accounts*, p. 217; Starr, ‘Sociology of official statistics’, pp. 18–20, 56–7; PrewittKenneth, ‘Public statistics and democratic politics’, in Alonso and Starr, Politics of numbers, pp. 261–274 .

45 Fioramonti, *How numbers rule*, pp. 210–13; Morse, *Indicators and indices*, pp. 180–3.

46 Cooley, ‘Emerging politics’, pp. 9, 21–3.

47

DavisKevin, KingsburyBenedict, and MerrySally Engle, ‘Introduction: global governance by indicators’, in Davis et al., Governance by indicators, p. 8 .

48
*Ibid.*, pp. 8–9; Starr, ‘Sociology of official statistics’, p. 40; Morse, *Indicators and indices*, p. 179.

49 Davis, Kingsbury, and Merry, ‘Indicators as a technology’, p. 87; Starr, ‘Sociology of official statistics’, pp. 40–52.

50 Starr, ‘Sociology of official statistics’, pp. 41, 53.

51 Fioramonti, *How numbers rule*, pp. 19–27.

52 Davis, Kingsbury, and Merry, ‘Introduction’, pp. 8, 15; Cooley, ‘Emerging politics’, pp. 20–1.

53 Davis, Kingsbury, and Merry, ‘Indicators as a technology’, pp. 93–4; Davis, Kingsbury, and Merry, ‘Introduction’, pp. 19–20.

54 Davis, Kingsbury, and Merry, ‘Indicators as a technology’, p. 30; Starr, ‘Sociology of official statistics’, p. 54.

55

BütheTim, ‘Beyond supply and demand: a political–economic conceptual model’, in Davis et al., Governance by indicators, pp. 32–34 ; Fisher, ‘From diagnosing under-immunization’, pp. 244–6.

56

DubinMartin, ‘The League of Nations Health Organization’, in Paul Weindling, ed., International health organisations and movements, 1918–1939, Cambridge: Cambridge University Press, 1995 .

57 Weindling, *International health organisations*, p. 135; WeindlingPaul, ‘The League of Nations Health Organization and the rise of Latin American participation, 1920–40’, História, Ciências, Saúde-Manguinhos, 13, 3, 2006, pp. 555–570 10.1590/s0104-5970200600030000217115525; Borowy, *Coming to terms*, p. 84.

58

KottSandrine and DrouxJoelle, eds., Globalizing social rights: the International Labour Organization and beyond, Basingstoke: Palgrave Macmillan, 2013 ; Weindling, *International health organisations*, p. 138.

59

KottSandrine, ‘Constructing a European social model: the fight for social insurance in the interwar period’, in Jasmin van Daele et al, eds., ILO histories: essays on the International Labour Organization and its impact on the world during the twentieth century, Bern: Peter Lang, 2010, pp. 173–195 .

60 Dubin, ‘League of Nations Health Organization’; ChorevNitsan, The World Health Organization between North and South, Ithaca, NY: Cornell University Press, 2012 .

61 ‘Constitution of the World Health Organization’, *American Journal of Public Health*, 36, 11, 1946, p. 1315, paras. c, f, p.

62

BuseKent, ‘Spotlight on international organizations: the World Bank’, Health Policy and Planning 9, 1, 1994, pp. 95–99 1013309910.1093/heapol/9.1.95; PrekerAlexander and FeachemRichard, ‘The role of the World Bank in facilitating health sector reform’, in Zuzana Feachem, Martin Hensher, and Laura Rose, eds., Implementing health sector reform in central Asia: papers from an EDI health policy seminar held in Ashgabat, Turkmenistan, June 1996, EDI Learning Resources Series, Washington, DC: The International Bank for Reconstruction, 1999, p. 144 .

63

KaaschAlexandra, Shaping global health policy: global social policy actors and ideas about health care systems, Basingstoke: Palgrave Macmillan, 2015 .

64

CarrollPeter and KellowAynsley, The OECD: a study of organisational adaptation, Cheltenham: Edward Elgar Publishing, 2011, pp. 1–2 .

65

OECD, Public expenditure on health, Studies in resource allocation no. 4, Paris: OECD, 1977 .

66 OECD archive, Paris, DEELSA/ELSA/WP1/M(95)1, ‘Working Party on Social Policy: summary record of the 14th meeting. Held at the Château de la Muette, Paris, 11–12 October 1995’, p. 3.

67

SwaroopS. and UemuraK., ‘Proportional mortality of 50 years and above: a suggested indicator of the component “health, including demographic conditions” in the measurement of levels of living’, Bulletin of the World Health Organization 17, 3, 1957, p. 442 PMC253761713500159.

68 Fioramonti, *Gross domestic problem*, pp. 24–32.

69

PEP (Political and Economic Planning), Report on the British health services, London: PEP, 1937, p. 391 .

70

ToozeAdam, Statistics and the German state: the making of modern economic knowledge, Cambridge: Cambridge University Press, 2001 .

71 Borowy, ‘World health’, pp. 94–5.

72

KottSandrine, ‘Dynamiques de l’internationalisation: l’Allemagne et l’Organisation Internationale du Travail (1919–1940)’, Critique Internationale, 3, 52, 2011, pp. 69–84 ; Kott, ‘Constructing a European social model’.

73

ChadwickEdwin, Report on the sanitary condition of the labouring population of Gt. Britain, Edinburgh: Edinburgh University Press, 1965 [1842], pp. 219–254 .

74

PinkerR., English hospital statistics 1861–1938, London: Heinemann, p. 1966 .

75 Borowy, ‘World health’, p. 126.

76

BorowyIris, ‘Counting death and disease: classification of death and disease in the interwar years, 1919–1939’, Continuity and Change, 18, 3, 2003, pp. 457–481 .

77

AmrithSunil, Decolonizing international health: India and Southeast Asia, 1930–65, Basingstoke: Palgrave Macmillan, 2006, pp. 9–11 , 23–4, 29–36.

78 League of Nations Health Committee, *Sessions 17–18, 1931*, ‘Fourth Meeting May 7th 1931. 10. Budget estimates for 1932’, pp. 42–3.

79 League of Nations Health Committee, *Sessions 19–20, 1932–33*, ‘Fifth Meeting October 15th. The crisis and its effects on public health’, pp. 82–3.

80 E.g. LNHO, International health yearbook 1927 (third year), Geneva: League of Nations, 1927 , title page.

81 Although statutory health insurance was relatively recent, returns variously included data on membership numbers or rates, claims incidence and duration, and revenue and expenditure. League of Nations Archive, Geneva (henceforth LONA), 8A/22711/19793, ‘1929 Health Year Book (Volume 6) Model statistical tables and various correspondence’, ‘Tableaux statistiques’; LONA, 8A/19793/19793, ‘1929 Health Year Book (Volume 6)’, ‘Suggestions to authors of reports for the International Health Year Book’.

82

SandRené, The advance to social medicine, London: Staples Press, 1952 .

83 See e.g. LONA, R 972/12B/57467/46868, anon. [Frank Boudreau(?)], ‘Proposals in regard to the further study of health insurance in relation to public health services from the point of view of preventive medicine’, February 1927.

84 LONA, R 968/12B/46868/46868, ‘Proposal of the Czechoslovak government for an enquiry into the health conditions of various countries’; ‘Notes on the health insurance inquiry’, March 1927.

85 Key officials in collaborative efforts were Rajchman’s assistant Frank Boudreau (LNHO) and Ferdinand Maurette (ILO). See, e.g., LONA, R 968/12B/46871/46848, letter from Albert Thomas, Director of the ILO, to Sir Eric Drummond, Secretary General of the League of Nations, October 1925. See also LONA, R 992, letter, Frank Boudreau, Health Section, LNHO to Dr J. Kuhn, Secretary of the Medical Association of Denmark, 30 March 1927.

86 LONA, R 992/12B/58852/57687, ‘Joint Commission of Experts for the study of the relationship between public health services and health insurance organisations: report of the first session’, 1927; LONA, R 992/12B/58852/57687, Sir George Newman, untitled draft speech, March 1927.

87 ILO, SI 21/5/2, ‘Mixed Commission of Public Health and Social Insurance. Sub-Commission of Social Medicine. General instructions for co-operation between social insurance organizations and social hygiene institutions in Czechoslovakia’, December 1929; LONA, R 970/12B/46868/48705, letter, Rajchman to Foramitti, 4 May 1925.

88 Borowy, *Coming to terms*; Borowy, ‘World health’.

89 Borowy, ‘World health’, pp. 91–4.

90 Dubin, ‘League of Nations Health Organization’, p. 57; Borowy, *Coming to terms*, pp. 34, 180.

91 LONA, 8A/22711/19793 ‘1929 Health Year Book (Volume 6): model statistical tables and various correspondence’, ‘Tableaux statistiques’; Borowy, *Coming to terms*, p. 182.

92

StoumanK. and FalkI. S., ‘Health indices: a study of objective indices of health in relation to environment and sanitation, Milbank Memorial Fund Quarterly, 15, 1, 1937, pp. 5–36 ; StoumanK. and FalkI. S., ‘An international system of health indices: a preliminary report’, American Journal of Public Health, 27, 1937, pp. 363–370 1801460710.2105/ajph.27.4.363PMC1563124.

93 WHO archive, Geneva, WHO/HS/19, WHO, ‘Expert Committee on Health Statistics: report of the first session’, 31 May 1949, p. 5.

94 WHO, ‘Expert Committee on Health Statistics: eighth report’, WHO Technical Report Series no. 261, Geneva: WHO, 1963, p. 8.

95

GillespieJames A., ‘Social medicine, social security and international health, 1940–60’, in Esteban Rodríguez Ocaña, ed., The politics of the healthy life: an international perspective, Sheffield: EAHMH, 2002 .

96 Brown, Cueto, and Fee, ‘World Health Organization’.

97

PackardRandall, The making of a tropical disease: a short history of malaria, Baltimore, MD: Johns Hopkins University Press, 2007 .

98 WHO, ‘Expert Committee on Health Statistics: report of the second session, Geneva, 18–21 April 1950’, WHO Technical Report Series no. 25, Geneva: WHO, 1950, p. 32.

99 WHO, ‘Expert Committee on Health Statistics: first session’; WHO, ‘Expert Committee on Health Statistics: third report’, WHO Technical Report Series no. 53, Geneva: WHO, 1952, pp. 16–17.

100

WHO, World Health Statistics Annual 1962, Geneva: WHO, 1965, pp. 24–25 .

101

GonzálezCarlos Luis, ‘The collection and use of health statistics in national and local health services’, Nineteenth World Health Assembly, Geneva: WHO, 6 May 1966, pp. 3–4 .

102

WHO, The second ten years of the World Health Organization 1958–67, Geneva: WHO, 1968, pp. 37–39 ; García GutiérrezJ., ‘Health planning in Latin America’, American Journal of Public Health, 65, 10, 1975, pp. 1047–1049 116369910.2105/ajph.65.10.1047PMC1776013.

103

HilleboeH., BarkhuusA., and ThomasW., Approaches to national health planning, Geneva: WHO, 1972, pp. 7, 15 5068040.

104 WHO, ‘Expert Committee on Health Statistics: thirteenth report. Statistics of health services and of their activities’, WHO Technical Report Series no. 429, Geneva: WHO, 1969, pp. 5–8.

105 WHO archive, Hist.Col WA 540 MW6 740R V2.

106

International Labour Office, The cost of medical care: Studies and reports 51, Geneva: International Labour Office, 1959 .

107
*Ibid.*, p. 156.

108
*Ibid.*, pp. 2, 156.

109

Abel-SmithB. and TitmussR. M., The cost of the National Health Service in England and Wales, Cambridge: Cambridge University Press, 1955, pp. 60–61 ; SheardSally, The passionate economist: how Brian Abel-Smith shaped global health and social welfare, Bristol: Policy Press, 2013 .

110

Abel-SmithB., Paying for health services: a study of the costs and sources of finance in six countries, Geneva: WHO, 1963 .

111

Abel-SmithB., An international study of health expenditure and its relevance for health planning, Geneva: WHO, 1967, pp. 46, 103 4973674.

112
*Ibid.*, pp. 88, 96.

113 WHO archive, WHO/OMC/1-27, Hernan Romero, ‘An approach to the problem of costs and financing of medical care services’, 8 August 1956.

114
*Ibid.*, p. 40.

115

APHA Task Force on Chile, ‘History of the health care system in Chile’, American Journal of Public Health, 67, 1, 1977, pp. 31–36 318809; Viveros-LongAnamaria, ‘Changes in health financing: the Chilean experience’, Social Science and Medicine, 22, 3, 1986, pp. 379–385 308351210.1016/0277-9536(86)90137-1.

116 APHA Task Force, ‘Health care system in Chile’, pp. 32–3.

117 OECD Health Policy Unit, ‘The state of implementation of the OECD manual: a system of health accounts (SHA) in OECD member countries’, 10 June 2001; UN, ‘Measurement of national income and the construction of social accounts: report of the Sub-Committee on National Income Statistics of the League of Nations Committee of Statistical Experts’, Studies and reports on statistical methods, Geneva: United Nations, 1947.

118 United Nations, *A system of national accounts and supporting tables*, Studies in methods no. 2, New York: United Nations, 1953. From 1982 the yearbook was renamed *National accounts statistics: main aggregates and detailed tables*.

119 Berman, ‘What can the U.S. learn?’; OECD Health Policy Unit, ‘State of implementation’; PommierPhilippe, ‘Social expenditure: socialization of expenditure? The French experiment with satellite accounts’, Review of Income and Wealth, 27, 4, 1981, pp. 373–386 ; GillionColin et al., Measuring health care 1960–1983, Social policy studies no. 2, Paris: OECD, 1985 .

120 Berman, ‘What can the U.S. learn?’

121 OECD, *Public expenditure on health*.

122
*Ibid.*; Kaasch, *Shaping global health policy*.

123

Abel-SmithB., ‘Foreword’, in Robert Maxwell, Health and wealth: an international study of health-care spending, Lexington, MA: Lexington Books, 1980, pp. xi–xii .

124 OECD, *Public expenditure on health*.

125 Gillion et al., *Measuring health care*.

126 OECD, ‘State of implementation’; OECD, Eurostat, and WHO, A system of health accounts 2011 edition, Paris: OECD Publishing, 2011 .

127

George Schieber and OECD, Financing and delivering health care: a comparative analysis of OECD countries, Social policy studies no. 4, Paris: OECD, 1987, p. 9 .

128

CuetoMarcos, ‘The origins of primary health care and selective primary health care’, American Journal of Public Health 94, 11, 2004, pp. 1864–1874 1551422110.2105/ajph.94.11.1864PMC1448553; NewellK., ed., Health by the people, Geneva: WHO, 1975 1181735.

129 Chorev, *World Health Organisation*.

130 Cueto, ‘Origins of primary health care’.

131

World Bank, World development report 1993: investing in health, Oxford: Oxford University Press, 1993 .

132

MurrayC. J. and AcharyaA., ‘Understanding DALYs’, Journal of Health Economics, 16, 1997, p. 707 10.1016/s0167-6296(97)00004-010176780.

133 World Bank, *World development report 1993*, p. iii.

134

de FerrantiDavid, Paying for health services in developing countries: an overview, Washington, DC: World Bank, 1985 ; World Bank, Financing health services in developing countries: an agenda for reform, Washington, DC: IBRD, 1987 3242740.

135 World Bank, *Financing health services*, p. 2.

136

MurrayC. J. and LopezA., ‘Progress and directions in refining the global burden of disease approach: a response to Williams’, Health Economics, 9, 1, 2000, p. 72 10.1002/(sici)1099-1050(200001)9:1<69::aid-hec493>3.0.co;2-i10694761; life tables from Japan provide the healthful norms on which calculation is based.

137 Adams, ‘Metrics of the global sovereign’, pp. 21–6.

138 Stouman and Falk, ‘Health indices’, pp. 9–10.

139 Swaroop and Uemura, ‘Proportional mortality’; Hilleboe, Barkhuus, and Thomas, *Approaches to national health planning*, pp. 54–6; SandersB., ‘Measuring community health levels’, American Journal of Public Health and the Nation’s Health, 54, 7, 1964, pp. 1063–1070 10.2105/ajph.54.7.1063PMC125492814157838; SullivanD. F., ‘A single index of mortality and morbidity’, HSMHA Health Reports, 86, 4, 1971, pp. 347–354 5554262PMC1937122; RosserR. M. and WattV. C., ‘The measurement of hospital output’, International Journal of Epidemiology, 1, 4, 1972, pp. 361–368 466908410.1093/ije/1.4.361; ZeckhauserR. and ShepardD., ‘Where now for saving lives?’, Law and Contemporary Problems, 40, 4, 1976, pp. 5–45 .

140 Murray and Acharya, ‘Understanding DALYs’, pp. 711–19.

141

SmithJeremy, Epic measures: one doctor, seven billion patients, New York: HarperCollins, 2015 .

142

WilliamsAlan, ‘Calculating the global burden of disease: time for a strategic reappraisal?’, Health Economics, 8, 1, 1999, pp. 1–8 1008213910.1002/(sici)1099-1050(199902)8:1<1::aid-hec399>3.0.co;2-b; SayersB. and FliednerT., ‘The critique of DALYs: a counter-reply’, Bulletin of the World Health Organization, 75, 4, 1997, pp. 383–384 9342898PMC2486969.

143

AnandS. and HansonK., ‘Disability-adjusted life years: a critical review’, Journal of Health Economics, 16, 6, 1997, pp. 685–702 1017677910.1016/s0167-6296(97)00005-2; ArensenT. and NordE., ‘The value of DALY life: problems with ethics and validity of disability adjusted life years’, British Medical Journal, 319, 1999, pp. 1423–1425 1057486710.1136/bmj.319.7222.1423PMC1117148.

144 Adams, ‘Metrics of the global sovereign’, 26–7.

145 Murray and Lopez, ‘Progress and directions’; Murray and Acharya, ‘Understanding DALYs’.

146 Murray and Acharya, ‘Understanding DALYs’, p. 717.

147

KnaulF. M. and FrenkJ., ‘Health insurance in Mexico: achieving universal coverage through structural reform’, Health Affairs, 24, 6, 2005, pp. 1467–1476 1628401810.1377/hlthaff.24.6.1467.

148

NavarroV., ‘Assessment of the *World health report 2000*
’, Lancet, 356, 2000, pp. 1598–1601 1107578910.1016/S0140-6736(00)03139-1; AlmeidaCelia et al., ‘Methodological concerns and recommendations on policy consequences of the *World health report 2000*
’, Lancet, 357, 2001, pp. 1692–1697 1142539410.1016/s0140-6736(00)04825-x.

149 Robert Helms, ‘Sick list: health care à la Karl Marx’, *Wall Street Journal Europe*, 29 June 2000.

150

McKeeMartin, ‘Measuring the efficiency of health systems’, British Medical Journal, 323, 2001, pp. 295–296 1149847310.1136/bmj.323.7308.295PMC1120916; BlendonR. J., KimM. and BensonJ. M., ‘The public versus the World Health Organization on health system performance’, Health Affairs, 20, 3, 2001, pp. 10–20 1158515710.1377/hlthaff.20.3.10; RobbinsA., ‘Book review’, Public Health Reports, 119, 2001, pp. 268–269 ; PedersenKjeld Møller, ‘The *World health report 2000*: dialogue of the deaf?’, Health Economics, 11, 2002, p. 94 10.1002/hec.69111921308.

151

WilliamsAlan, ‘Science or marketing at WHO? A commentary on “World health 2000”’, Health Economics, 10, 2001, pp. 93–100 1125204110.1002/hec.594.

152 Knaul and Frenk, ‘Health insurance in Mexico’.

153

ZhangXiulan et al., ‘Advancing the application of systems thinking in health: managing rural China health system development in complex and dynamic contexts’, Health Research Policy and Systems, 12, 44, 2014, p. 5 2515972610.1186/1478-4505-12-44PMC4245849.

154 Barack Obama, ‘Remarks by the President to a Joint Session of Congress on Health Care September 9, 2009’, https://web.archive.org/web/20100308000433/https://www.whitehouse.gov/the-press-office/remarks-president-a-joint-session-congress-health-care (consulted 22 June 2017).

155 McKee, ‘*World health report 2000*’.

156

SaltmanR. B. and Ferroussier-DavisO., ‘The concept of stewardship in health policy’, Bulletin of the World Health Organization, 78, 6, 2000, pp. 732–739 10916910PMC2560782.

